# REG*γ* Contributes to Regulation of Hemoglobin and Hemoglobin *δ* Subunit

**DOI:** 10.1155/2017/7295319

**Published:** 2017-07-16

**Authors:** Qiuhong Zuo, Shanshan Cheng, Wenxiang Huang, Muhammad Zeeshan Bhatti, Yanyan Xue, Yuanyuan Zhang, Bianhong Zhang, Lei Li, Lin Wu, Junjiang Fu, Jiwu Chen, Xiaotao Li

**Affiliations:** ^1^Shanghai Key Laboratory of Regulatory Biology, Shanghai Key Laboratory of Brain Functional Genomics (Ministry of Education), Institute of Biomedical Sciences, School of Life Sciences, East China Normal University, 500 Dongchuan Road, Shanghai 200241, China; ^2^Department of Infectious Diseases, The First Affiliated Hospital of Chongqing Medical University, Chongqing 400016, China; ^3^Key Laboratory of Epigenetics and Oncology, the Research Center for Preclinical Medicine, Southwest Medical University, Sichuan 646000, China; ^4^Department of Molecular and Cellular Biology, Dan L. Duncan Cancer Center, Baylor College of Medicine, One Baylor Plaza, Houston, TX 77030, USA

## Abstract

Hemoglobin (Hb) is a family of proteins in red blood cells responsible for oxygen transport and vulnerable for oxidative damage. Hemoglobin *δ* subunit (HBD), a member of Hb family, is normally expressed by cells of erythroid lineage. Expression of Hb genes has been previously reported in nonerythroid and hematopoietic stem cells. Here, we report that Hb and HBD can be degraded via REG*γ* proteasome in hemopoietic tissues and nonerythroid cells. For this purpose, bone marrow, liver, and spleen hemopoietic tissues from *REGγ^+/+^* and *REGγ^−/−^* mice and stable REG*γ* knockdown cells were evaluated for the degradation of Hb and HBD via REG*γ*. Western blot and immunohistochemical analyses exhibited downregulation of Hb in REG*γ* wild-type mouse tissues. This was validated by dynamic analysis following blockade of de novo synthesis of proteins with CHX. Degradation of HBD only occurred in REG*γ* WT cells but not in REG*γ*N151Y, a dominant-negative REG*γ* mutant cell. Notably, downregulation of HBD was found in HeLa shN cells with stimulation of phenylhydrazine, an oxidation inducer, suggesting that the REG*γ* proteasome may target oxidatively damaged Hbs. In conclusion, our findings provide important implications for the degradation of Hb and HBD in hemopoietic tissues and nonerythroid cells via the REG*γ* proteasome.

## 1. Introduction

Hemoglobin (Hb) is one of the most abundant proteins in the human body and the major component of erythrocytes. A growing number of studies describe the human red blood cells (RBCs) life span in the circulatory system as approximately 100–120 days. Under the physiology condition, it is estimated that everyday, about 1/120 RBCs are generated and same number of RBCs attempt suicidal cell death or erythrosis in the human body. Eryptosis is characterized by cell shrinkage, cell membrane bleeding, and cell membrane phospholipids scrambling with phosphatidylserine exposure at the cell surface. In this regard, eryptosis is stimulated by an increase in cytosolic Ca^2+^ activity, ceramide, hyperosmotic shock, oxidative stress, energy depletion, hyperthermia, and a wide verity of xenobiotics and endogenous substances [1, 2]. These eryptosis factors also participate in the Hb damage of RBCs as well as in various hemoglobinopathies, which have been observed in Hb damage. Similarly, genetic factors have been implicated in hemoglobinopathies. In view of these similarities to the physiological and pathological roles of Hb, diminishing the metabolic distribution, the cells have developed a more vulnerable degradation system through which one can recognize damaged or abnormal Hb. Beside these effects, the degradation and removal of damaged/abnormal Hb in RBCs are also assessed by the proteasomal system [3–5]. Research into the molecular mechanism of protein damage of RBCs in human cell line is more fascinating.

Indeed, recent evidence suggests that proteasome exists in various forms and its activity is modulated by multiple activators including 19S, 11S (REG), and PA200. These proteasomes are composed of a 20S core, with three distinct catalytic sites, and proteasomal activators [6, 7]. Furthermore, as it was reported that misfiled protein or short-lived regulatory proteins are mostly degraded in an ATP- and ubiquitin-dependent pathway by the 26S proteasome, composing of the 20S “core” and the 19S regulator cap. In addition, some abnormal proteins can be degraded in an ATP- and ubiquitin-independent pathway by the 11S proteasome, which consists of 20S “core” proteasome and 11S regulator cap, among which REG*γ* emerges as a biologically important regulatory protein [8–11]. The REG (11S) family protein members include REG*α*, REG*β*, and REG*γ*, which contribute to almost 35% identical amino acids. The 19S activator contains an ATPase subunit, which degrades proteins in an ATP- and ubiquitin-dependent manner, when bind to the 20S proteasome [7]. In contrast, the REG family mediates protein degradation in an ATP- and ubiquitin-independent manner [6, 7]. Growing amount of evidence suggests that 20S and 26S proteasomes are present in RBCs. These proteasomes are biologically active, with higher 20S proteasome activity than 26S proteasomal activity [3–5]. Therefore, the proteasome activators such as 19S and 11S regulators and their regulation are importance for the homeostasis of RBCs [3, 4]. In fact, Hb degradation of RBCs by proteasome systems is thought to play an important role biochemically and clinically.

In addition, recent studies have shown that REG*α*/*β* and 20S proteasome play a role in the oxidized Hb degradation [12]. The precise role of REG*γ* in the degradation of Hb is still unclear. Despite that human mature RBCs consisting of HbA (*α*2*β*2; 95–98%), HbA2 (*α*2*δ*2; 1.5–3.5%), and HbF (*α*2*γ*2; <1%) are well documented for Hb subunits *α* chain and/or *β* chain, less studies focus on degradation of Hb *δ* subunit (HBD). Therefore, we have investigated the role of REG*γ* proteasome in the degradation of Hb and HBD. Our data provides evidence that REG*γ* is a regulator for Hb and HBD degradation. The noncanonical proteasomal degradation system (REG*γ*) identified in this study will shed light on in-depth investigation and understanding the functional pathway of Hb and HBD degradation.

## 2. Materials and Methods

### 2.1. Materials

Dulbecco's modified Eagle's medium (DMEM), fetal bovine serum (FBS), and antibiotics (penicillin and streptomycin sulfate) were purchased from Life Technologies Inc. Phenylhydrazine (PHZ) was purchased from Linfeng Chemical Reagent Company (Shanghai, CN). Cycloheximide (CHX) was purchased from Sigma (St. Louis, MO). Anti-REG*γ* and anti-*β*-actin antibodies were purchased from Abmart; anti-Hb and human HBD antibodies were purchased from Santa Cruz Biotech Inc.; anti-p21 and anti-Smurf 1 antibodies were purchased from BD Biosciences Inc. (USA). Second antibodies were purchased from LI-COR Biosciences (USA) and/or Abmart.

### 2.2. Cell Culture

The stable REG*γ* knockdown HeLa (HeLa shN and shR) cells were previously generated in our laboratory [9, 13, 14]. Cells were routinely grown in Dulbecco's modified Eagle's medium (DMEM) supplemented with 10% FBS and antibiotics (100 mg/ml penicillin and 50 mg/ml streptomycin sulfate) at 37°C with 5% CO_2_ atmosphere. The HeLa cells were seeded at 40–50% confluences and split at least twice a week.

### 2.3. Construct Subcloning (pSG5-HBD Plasmid)

The required HBD strain was screened from the library. Taking the human cDNA as a template, RT-PCR amplification was preformed and then cloned into pSG5 vector with HA tag. Briefly, HBD was generated by using the primers HBD-XholI-Forward: 5′-CGACTCGAGATGGTGCATCTGACTCCTGAG-3′ and HBD-NotI-Reverse: 5′-GAGCGGCCGCTCAATGGTACTTGTGAGCC-3′. PCR analysis was performed with the above specific primers on a PCR Amplifier (Bio-Rad, USA). Each PCR reaction contained 2 *μ*l cDNA-HBD, 1 *μ*l forward primer, 1 *μ*l reverse primer, 1 *μ*l dNTPs, 0.5 *μ*l pFu DNA polymerase, 10× pFu buffer, and 17 *μ*l QH_2_O (PCR-grade) in a 25 *μ*l reaction volume. The optimized assay conditions were 95°C for 5 min followed by 32 cycles of amplification (95°C for 45 sec, 52°C for 1 min, and 72°C for 60 sec) and a final extension step at 72°C for 10 min. The amplified product was subjected to agarose gel electrophoresis and the target gene HBD cDNA was extracted by QIAquick Gel Extraction kit (Tiangen Biotech Co. Ltd.) according to the manufacturer's specifications. Then, HBD cDNA was inserted into HA-pSG5 plasmid. The constructs were verified by sequencing.

### 2.4. Cell Transfection

HeLa cells at 70% confluence were transiently transfected with the plasmids HA-pSG5-HBD, FRT-REG*γ*wt, and FRT-REG*γ*N151Y, using Lipofectamine 2000 transfection reagent (Invitrogen) in accordance with the manufacturer's instructions. The empty vectors such as HA-pSG5-vector and FRT-vector were transfected as controls. Cells were incubated at 37°C in CO_2_ incubator; after 6–8 h later, 10% serum growth medium was added to the transfection mixture. Cell extracts were evaluated via Western blot for REG*γ* and HBD protein expression at 48 h posttransfection.

### 2.5. Oxidation Analysis

Oxidation analysis was performed for HBD degradation in HeLa shN and shR cells. Briefly, HeLa cells at 70% confluence were transiently transfected with HA-pSG5-HBD plasmid in incubation for 48 h. HeLa shN and shR cells were treated with (5 mM) phenylhydrazine (PHZ) for 0, 1, and 2 h in a time-dependent manner.

### 2.6. Western Blot Analysis

Western blot was performed using the standard method; cells were cultured to confluence in 6-well plates. The samples were collected with or without plasmids transfection or oxidant reagents; cells were washed with phosphate-buffered saline (PBS) and treated with protein extraction buffer (50 mM Tris-HCl, pH 7.5, 150 mM NaCl, 1% NP-40, 1 mM EDTA, 20 mM Na_3_VO_4_, and 1 mM PMSF). The sample concentration was measured by BCA protein assay kit (Pierce, USA). Protein samples were subjected to electrophoresis in 10% SDS polyacrylamide gel (SDS-PAGE). Separated proteins were electro blotted to nitrocellulose membranes (Bio-Rad), and the blot was blocked for 1 h at room temperature with blocking buffer (0.1% PBST with 5% fat-free dried milk powder). The blot was then incubated with primary antibodies (1 : 1000 dilutions) at 4°C overnight. The blot was washed with 0.1% TBST 3 times and incubated with secondary antibodies (mouse, rabbit) (1 : 5000 dilution) for 1 h. The blot was washed again 3 times and exposed to Odyssey LI-COR scanner.

### 2.7. Immunofluorescence (IF) Staining

Sterile slides were inserted into 24-well plates; pretransfected HeLa cells were plated into each well at a concentration of 5 × 10^4^/ml and incubated at 37°C with 5% CO_2_ for 24 h. Next, 4% paraformaldehyde was added for 10 min at room temperature for fixation, permeabilized with 0.25% Triton X-100. Then, cells were washed with PBS, three times for 1 min each time, and blocked with 1% BSA for 1 h at room temperature. Subsequently, anti-REG*γ* and anti-HBD antibodies were incubated at 4°C overnight. Cells were incubated with secondary antibodies Alexa Fluor 488-conjugated goat anti-rabbit IgG and Alexa Fluor 594 goat anti-mouse IgG (Abmart) for 1 h, and nuclei were stained with 4',6-diamidino-2-phenylindole (DAPI). Images of the cells were captured under fluorescence microscopes (LI-COR Bioscience Co.) and analyzed by Image-Pro Plus 6.0 software.

### 2.8. Animal Care

*REG*γ*^−/−^* mice with C57BL/6 genetic background were acquired from Dr. John J. Monaco (University of Cincinnati College of Medicine, Cincinnati) [15]. Our laboratory maintained *REGγ^+/−^* mice and kept intercrosses between males and females for generation of *REGγ^+/+^* and *REGγ^−/−^* mice. Genotyping of *REGγ^+/+^* and *REGγ^−/−^* mice was carried out by PCR analysis of genomic DNA as described [15]. All mice were bred in the Animal Core Facility at 20–26°C with 40–70% humidity and 12 h light/dark cycle (07:00–19:00). Standard rodent diet and water were provided ad libitum throughout the study. There were no more than five animals per cage. All mice were sacrificed after feeding for 1 week or 2 weeks. Liver, spleen, and bone marrow tissues were collected form all mice at the same time and paraffin-embedded sections (4 *μ*m thickness) or tissue homogenates were prepared. All procedures were carried out in accordance with the guidelines of the National Institutes of Health Guide for the Care and Use of Laboratory Animals. All the animal experiments were performed according to the approval of the Animal Care Committee of East China Normal University.

### 2.9. Immunohistochemistry Analysis

Paraffin-embedded sections of liver and spleen tissues from *REGγ*^+/+^ mice and *REGγ^−/−^* mice were used to perform IHC staining. Tissue sections were deparaffined with xylene and dehydrated with sequential washing of 100%, 95%, 85%, 75%, and 50% ethanol. Endogenous peroxidase activity was quenched using 3% H_2_O_2_ in methanol for 10 min and then washed three times in PBS. Antigen retrieval was achieved using a water bath heating in citric acid retrieval solution, 0.01 M, pH 7.6, at 100°C for 30 min, followed by cooling at room temperature. Slides were then incubated with anti-HBD antibody (1 : 300) dilutions and anti-REG*γ* antibody (1 : 500) dilutions at 4°C overnight. Next, the slides were rinsed three times in PBS and incubated in biotin-labeled rabbit anti-rabbit secondary antibodies for 10 min at room temperature. After washing three times with PBS, the staining was performed using diaminobenzidine developing solution. The sections were counter-stained with hematoxylin. We compared IHC staining between *REGγ^+/+^* and *REGγ^−/−^* mice liver tissues by percentage of intensity of staining to estimate the changes of HBD expression. Similarly, the difference of HBD expression were compared between *REGγ^+/+^* and *REGγ^−/−^* mice spleen tissues. The specimens were then mounted and examined under a light microscope (Model BX-61; Olympus Corp., Tokyo, Japan).

### 2.10. RT-PCR Analysis

The experiments were conducted with 2-week-old *REGγ^+/+^* and *REGγ^−/−^* mice housed in specific pathogen-free conditions and handled according to the ethical and scientific standards by the animal center in the institute (Minhang Laboratory of Animal Center at East China Normal University). Total RNA from bone marrow, spleen, and liver tissues (*REGγ^+/+^* and *REGγ^−/−^* mice) were isolated using TRIzol (Invitrogen) following the manufacturer's protocol. Briefly, 1-2 *μ*g of total RNA was reverse-transcribed to cDNA, the final reaction system of 20 *μ*l which contains 1 *μ*l Random Primer, 1-2 *μ*g RNA, 1 *μ*l 10 mM dNTP, and DEPC water. The RT-PCR reaction was performed at 25°C for 10 min, 37°C for 50 min, and 70°C for 15 min. All cDNA samples were stored at −80°C until further analysis. Primer sequences are described as follows: CD163-sense: 5′-TTTGTCAACTTGAGTCCCTTCAC-3′; CD163-antisense: 5′-TCCCGCTACACTTGTTTTCAC-3′; 18S-sense 5′-GGACACG GACAGGATTGA CA-3′; 18S-antisense 5′-GACATCTAAGGGCATCACAG-3′. The primers were synthesized by BioSune Biotechnology (Shanghai) Co. Ltd. The 2 *μ*l of reverse-transcribed cDNA was subjected to quantitative real-time PCR using master mix with SYBR-green (TOYOBO) and the Mx3005P quantitative RT-PCR system (Stratagene). Each reaction system contains 1 *μ*l primer mix, 1 *μ*l cDNA, 7.96 *μ*l miliQ water, 10 *μ*l SYBR green, 0.04 *μ*l 50× ROX to a final volume of 20 *μ*l. Each experiment was performed in duplicates and repeated thrice. The data was normalized to 18S mRNA. The delta threshold cycle value (∆Ct) was calculated using the formula ∆Ct = Ct gene − Ct control. The fold change was calculated as 2^−∆Ct^.

### 2.11. Statistical Analysis

All data is expressed as means ± SD. One-way analysis of variance (ANOVA) or Student's *t*-test was conducted to find out the significance of variations. A probability level of *p* < 0.05 was selected, indicating statistical significance. All the experiments were repeated in three independent experiments.

## 3. Results

### 3.1. REG*γ* Deficiency Promotes Hb in Mouse Tissues

**Figure 1:**
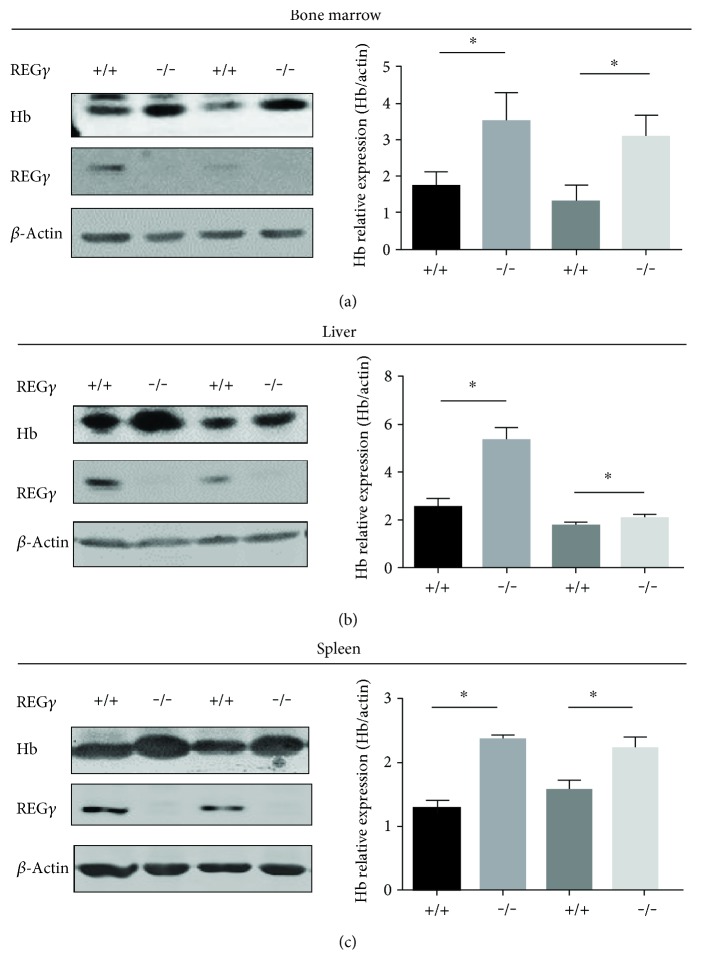
REG*γ*-mediated Hb degradation in multiple hemopoietic tissues. (a) Hb levels in bone marrow, (b) liver, and (c) spleen tissues from two different *REGγ^+/+^* and *REGγ^−/−^* mice. Protein was extracted and analyzed using Western blot against anti-Hb and anti-REG*γ* antibodies. *β*-Actin was use as internal control. The quantification analysis was conducted and shown as a graph. Left: Western blot. *n* = 3 represents the number of mice in each genotype. All the mice were sacrificed at age of 1-2 weeks old. Right: quantification analysis. Data are presented as means ± SEM from three independent experiments. ^∗^*p* < 0.05 versus control.

The REG family member includes REG*α* and REG*β*, as well as 20S proteasome has been shown to induce oxidative Hb degradation in murine embryonic fibroblasts cells [12]. Therefore, we determined whether the other members of REG (11S) proteasome family activator, REG*γ*, might regulate degradation of oxidized Hb or Hb in REG*γ* wild-type and knockout mice tissues. We investigated the roles of REG*γ*-mediated regulation of Hb degradation in *REGγ^+/+^* and *REGγ^−/−^* mice tissues (1-2-week-old mice), including the bone marrow, liver, and spleen, since these tissues are associated with removal of aged erythrocytes and clearance of oxidized Hb. We observed that *REGγ^+/+^* bone marrow tissues exhibited significant downregulation of Hb protein levels by Western blot analysis. In contrast, upregulation of Hb expression was noted in *REGγ^−/−^* bone marrow tissues (Figure 1(a)). Interestingly, as shown in Figures 1(b) and 1(c), REG*γ* wild-type tissues from the liver and spleen showed significant decreased Hb protein expression, while REG*γ* knockout mice tissues promoted Hb in both liver and spleen tissues. These results suggest that REG*γ* plays an important role in the Hb degradation of hemopoietic tissues.

**Figure 2:**
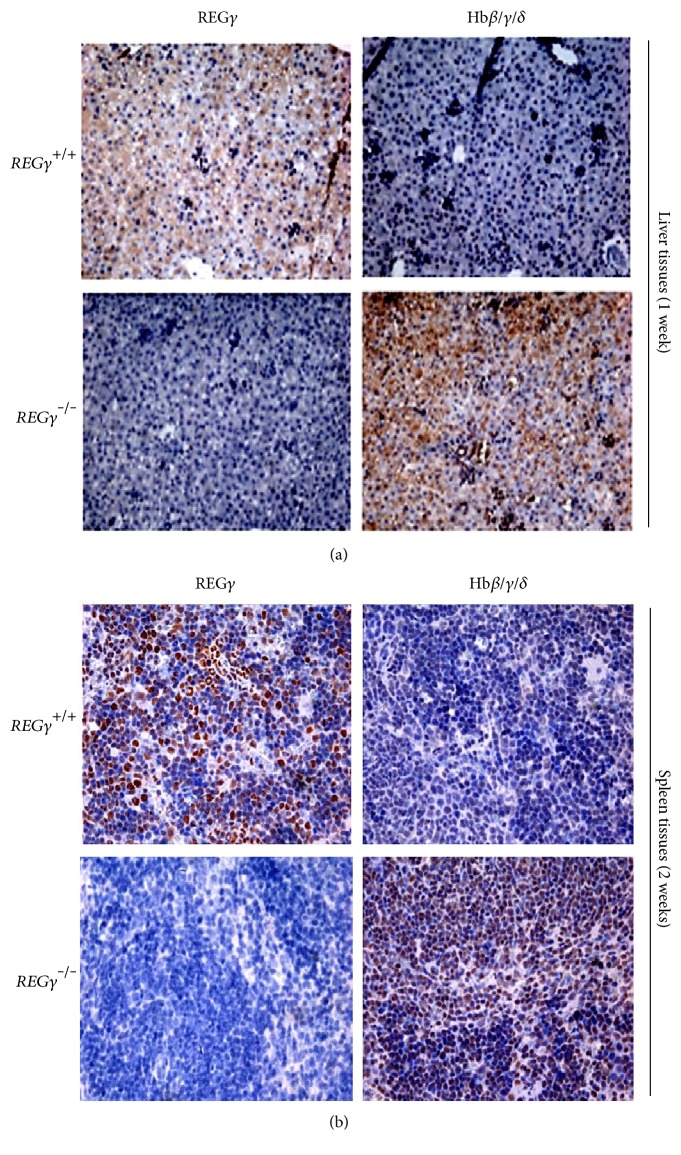
Negative correlation between REG*γ* and Hb in liver and spleen tissues from REG*γ* wild-type and knockout neonatal mice. Immunohistochemical staining was performed with anti-REG*γ* and anti-Hb *β/γ/δ* antibodies in *REGγ^+/+^* and *REGγ^−/−^* mice tissues. The tissues for REG*γ* positive and the Hb positive showed brown granules, respectively. (a) Liver tissues were analyzed for immunohistochemical analysis from one-week-old mice. (b) Spleen tissues from *REGγ^+/+^* and *REGγ^−/−^* mice (2 weeks old) were subjected to immunohistochemical analysis. The results of the representative figures were reproduced in three independent experiments.

Based on our observation of Hb protein downregulated by REG*γ* in *REGγ^+/+^* and *REGγ^−/−^* mice tissues, we further explored the relationship of REG*γ* and Hb by performing immunohistochemical staining analysis. We identified liver specimens of one-week-old *REGγ^+/+^* and *REGγ^−/−^* mice with anti-REG*γ* and anti-Hb*β/γ/δ*, as shown in Figure 2(a). Results indicated that *REGγ^+/+^* liver tissues expressed a high level of REG*γ* and a low level of Hb. Furthermore, we analyzed spleen tissues from 2-week-old *REGγ^+/+^* and *REGγ^−/−^* mice. We observed a high level of REG*γ* and a low level of Hb in *REGγ^+/+^* spleen tissues, whereas *REGγ^−/−^* spleen tissues exhibited a high level of Hb and a low level of REG*γ* (Figure 2(b)). These results indicate a potentially inverse correlation between REG*γ* and Hb.

### 3.2. REG*γ* Promotes CD163 Expression in Mice Tissues

**Figure 3:**
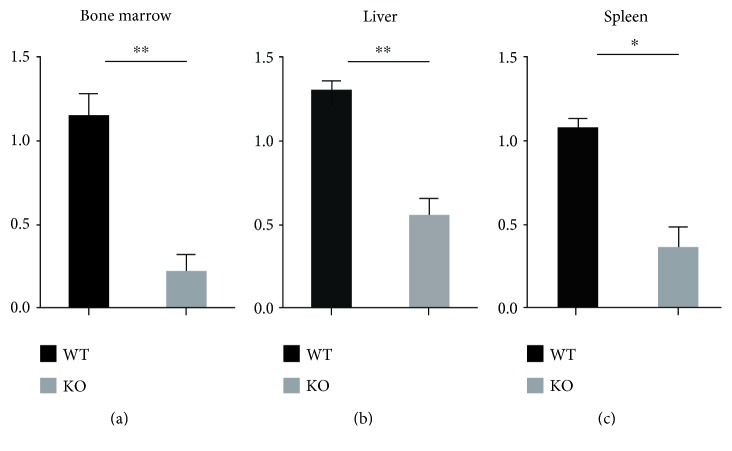
REG*γ* promotes CD163 expression in mice tissues. mRNA expression of CD163 was measured by qRT-PCR in (a) bone marrow, (b) liver, and (c) spleen tissues from *REGγ^+/+^* and *REGγ^−/−^* mice. Data was derived from independent experiments with *n* = 6 + 6 (*REGγ^+/+^* and *REGγ^−/−^* mice). Error bars represent mean ± SD; ^∗^*p* < 0.05; ^∗∗^*p* < 0.01 versus control, as determined by Student's *t*-test.

CD163 is a member of the cysteine-rich scavenger receptor family, and has been identified as an Hb scavenger receptor, mediating uptake of Hb in the circulation system. To elucidate the mechanism underlying interaction between REG*γ* and CD163 in bone marrow, liver, and spleen tissues from *REGγ^+/+^* and *REGγ^−/−^* mice, we evaluated the mRNA expression of CD163 by qRT-PCR analysis. We detected a significant increase of CD163 (4.3-fold change) in REG*γ* bone marrow tissues as compared to REG*γ* knockout mice tissues. These results promoted us to test the mRNA level of CD163 in liver and spleen tissues from *REGγ^+/+^* and *REGγ^−/−^* mice. We found attenuated mRNA levels of CD163 in liver (1.4-fold change) and spleen (2.2-fold change) tissues from *REGγ^−/−^* mice, compared with those from *REGγ^+/+^* mice by qRT-PCR analysis (Figure 3). Overall, CD163 expression was dramatically higher in bone marrow tissues in comparison with liver and spleen tissues. Taken together, this data demonstrated that REG*γ* mediates a stimulatory effect on Hb degradation through upregulation of CD163 activity in these tissues.

### 3.3. Hemoglobin *δ* Subunit (HBD) Regulation in HeLa Cells

**Figure 4:**
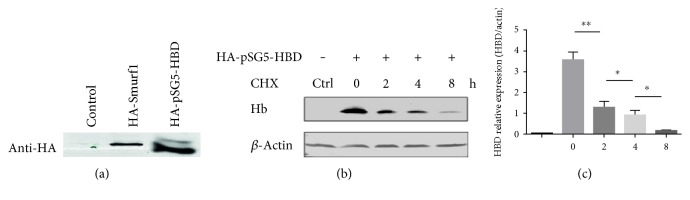
Regulation of endogenous HBD in HeLa cells. (a) HeLa cells were transfected with HA-pSG5-HBD plasmid (1 *μ*g/ml) and HA-Smurf1 (1 *μ*g/ml) as a positive control, using Lipo2000 transfection reagent for 72 h incubation. Total protein extracts were analyzed by Western blot against anti-HA antibody. (b) CHX assay for endogenous degradation. HeLa cells transfected with HA-pSG5-HBD. After 64 h incubation, cells were treated 100 *μ*g/ml of CHX for 0, 2, 4, 6, and 8 h. The total protein extracts were subjected to Western blot analysis. *β*-Actin was used as loading control. (c) Quantification of CHX-treated Western blot results expressed as the means ± SEM. ^∗^*p* < 0.05; ^∗∗^*p* < 0.01 versus control.

HBD typically accounts for approximately 10–30% of total hemoglobin in definitive erythrocytes, and available evidence suggests that it is generally characterized by elevated O_2_-binding properties, regulatory changes in intraerythrocytic mechanism. To clarify the function of REG*γ* in HBD degradation, nonblood cells were used for mechanistic studies since they had no endogenous HBD. HBD plasmid was constructed in pSG5 vector with HA tag for the overexpression of HBD, and HA-Smurf1 plasmid was used as control for expression of exogenous DNA. HA-pSG5-HBD was transfected with Lipo2000 reagent for 72 h in HeLa cells and evaluated by Western blot analysis. Results indicated that a significantly increased protein level of HBD was exhibited by the HA-pSG5-HBD-transfected HeLa cells (Figure 4(a)). To further investigate the role of REG*γ*-mediated degradation of HBD, HeLa cells were treated with CHX (100 *μ*g/ml), an inhibitor for de novo protein synthesis, in a time-dependent manner. Interestingly, we found that CHX treatment dramatically displayed the degradation effects of REG*γ* on HBD in a time-dependent manner, supporting the hypothesis that HBD is degraded by the REG*γ* proteasome (Figures 4(b) and 4(c)). These results indicate that REG*γ* regulates HBD protein stability in HeLa cells.

### 3.4. REG*γ*-Mediated Degradation of HBD

**Figure 5:**
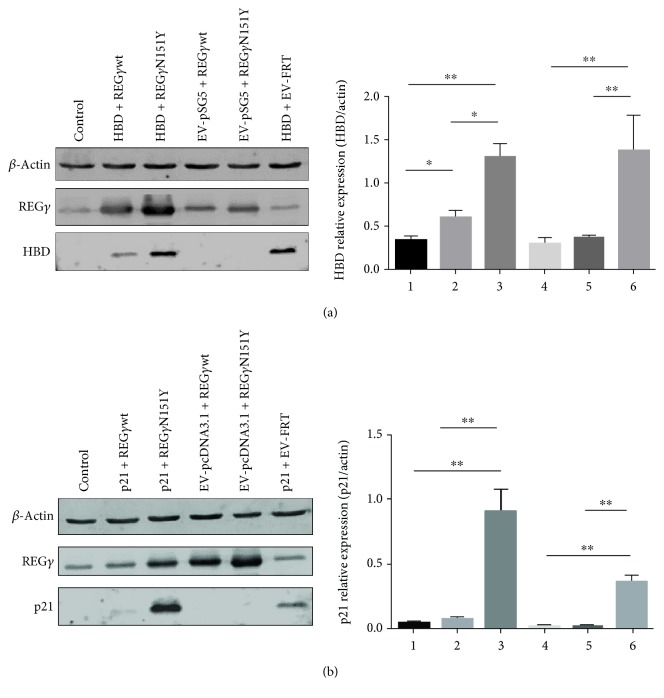
REG*γ*-mediated degradation of HBD in HeLa cells (a). Cells were transiently transfected with HA-pSG5-HBD (2 *μ*g), FRT-REG*γ*wt (1 *μ*g), FRT-REG*γ*N151Y (1 *μ*g), HA-pSG5 vector (2 *μ*g), and FRT vector (1 *μ*g) using Lipo2000 transfection reagent for 72 h incubation. Protein expression was detected against anti-REG*γ*, anti-HBD, and anti-*β*-actin antibodies. Nontransfected HeLa cells were used as a control. (b) HeLa cells were transfected with pcDNA3.1-p21 (2 *μ*g) FRT-REG*γ*wt (1 *μ*g), FRT-REG*γ*N151Y (1 *μ*g), pcDNA3.1 vector (2 *μ*g), and FRT vector (1 *μ*g) using Lipo2000 transfection reagent for 72 h incubation. Protein expressions of indicated antibodies were determined by Western blot analysis. Nontransfected HeLa cells were used as controls. *β*-Actin was used as loading control. The quantification analysis was conducted and shown as a graph. Left: Western blot. Right: quantification analysis. Data is presented as means ± SEM. ^∗^*p* < 0.05; ^∗∗^*p* < 0.01 versus control.

To elaborate REG*γ*-dependent degradation of HBD, we constructed HA-pSG5-HBD, FRT-REG*γ*wt, and FRT-REG*γ*N151Y plasmids, with empty vectors HA-pSG5 and FRT as controls. The pcDNA3.1-p21 plasmid was used as a positive control, since p21 is a known target of REG*γ* (Figure 5(b)). HBD level was significantly lower in the presence of overexpression of REG*γ*wt, whereas HBD was much higher when the mutant REG*γ*N151Y was exogenously expressed (Figure 5(a)). Notably, the dominant-negative mutant REG*γ*N151Y (with a single amino acid mutation “activation ring” of REG*γ*) abrogated the proteasome activity, although the binding ability to the proteasome is maintained [15, 16]. As a positive control, p21 showed similar patterns to HBD in the presence of either WT or mutant REG*γ* (Figure 5(b)). Taken together, these results demonstrate that REG*γ* is a novel mediator for the degradation of HBD.

### 3.5. Cellular Location and Expression of HBD in HeLa Cells

**Figure 6:**
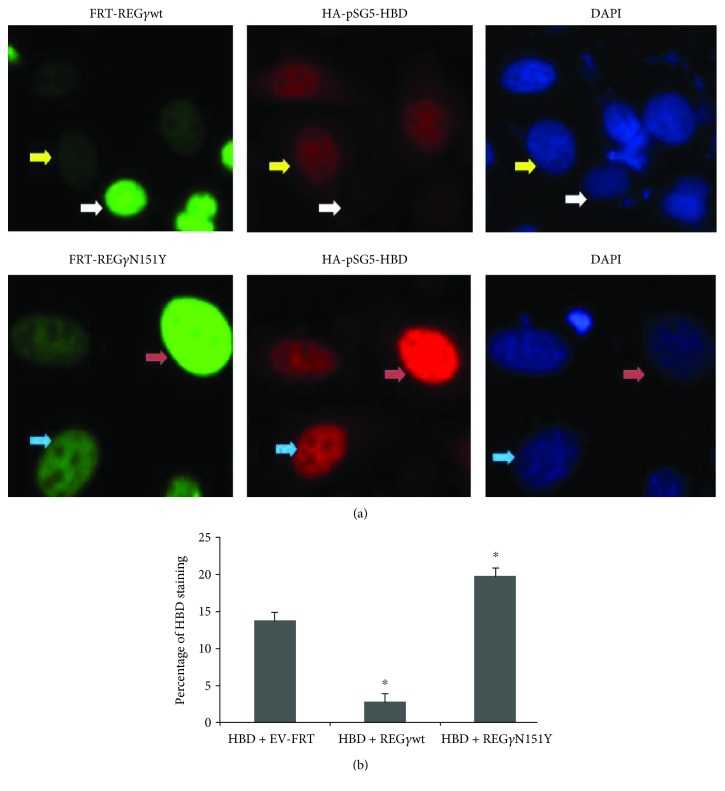
Cellular location and expression of HBD in HeLa cells. (a) Cells were transiently transfected with HA-pSG5-HBD (2 *μ*g) + FRT-REG*γ*wt, HA-pSG5-HBD (2 *μ*g) + FRT-REG*γ*N151Y (1 *μ*g), and HA-pSG5-HBD (2 *μ*g) + HA-pSG5 vector (2 *μ*g). Cells were fixed and immunostained with anti-REG*γ* (green color) and anti-HBD (red color). Cell nuclei were stained with DAPI staining (blue color). Scale bar: 50 *μ*m. (b) Percentage of HBD expression in HeLa cells with HA-pSG5-HBD + FRT-REG*γ*wt, HA-pSG5-HBD + FRT-REG*γ*N151Y, and HA-pSG5-HBD + HA-pSG5 vector. Each data represents the means of three independent experiments. Bars are the standard errors, *n* = 300. Significance was determined by Student's *t*-test. ^∗^*p* < 0.05 versus control.

Next, we sought to examine the effect of REG*γ* and HBD on cellular localization and expression by immunofluorescence (IF) staining. HA-pSG5-HBD + FRT-REG*γ*wt and HA-pSG5-HBD + FRT-REG*γ*N151Y plasmids were transiently transfected into HeLa cells, respectively. Results of immunostaining indicated that co-transfection of HA-psG5-HBD (red) and FRT-REG*γ*wt (green) significantly inhibited the expression of HBD in HeLa cells, while FRT-REG*γ*N151Y (green) expressing cells had stabilized expression of HBD (Figure 6(a)). These results demonstrated a critical role of REG*γ*-mediated regulation of HBD in vitro. Further, quantification of HBD positive cells/total cells expression was quantitated for statistical analysis. We observed that HeLa cells with cotransfection of HA-pSG5-HBD and FRT-REG*γ*wt exhibited reduction in HBD repression. In contrast, cotransfection of HA-pSG5-HBD and FRT-REG*γ*N151Y in HeLa cells displayed elevation in HBD expression level as compared to HA-pSG5-HBD and HA-pSG5 vector-transfected cells (Figure 6(b)). Thus, the immunofluorescence study provides evidence for the relationship between REG*γ* and HBD.

### 3.6. REG*γ* Promotes HBD Degradation under Oxidative Stress

**Figure 7:**
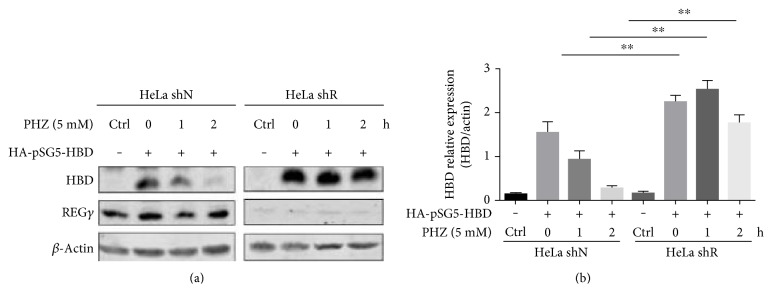
REG*γ* promotes HBD degradation under oxidative stress. HeLa shN and HeLa shR cells were transfected with or without HA-pSG5-HBD plasmid. Cells were treated with a 5 mM concentration of PHZ for 0, 1, and 2 h. Oxidative stress via PHZ stimulation on HBD in the presence or absence of REG*γ* was measured by Western blotting against anti-HBD and anti-REG*γ* antibodies. *β*-Actin was used as internal control. (b) Quantification of HA-pSG5-HBD- and PHZ-treated Western blot results expressed as the means ± SEM. ^∗∗^*p* < 0.01 versus control.

Reactive oxygen species- (ROS-) mediated Hb degradation increase the oxidative damage of erythrocytes via an ATP- and ubiquitin-dependent manner [17, 18]. Interestingly, proteasome activator REG*γ* has been reported to promote degradation in an ATP- and ubiquitin-independent manner [6, 7] and to mediate oxidative Hb degradation [12]. To elucidate the underlying mechanism of REG*γ*-mediated Hb degradation via oxidative HBD, we utilized stable knockdown REG*γ* HeLa cells (HeLa shN and shR cells) previously generated in our laboratory. We determined the oxidative effect of HBD degradation in HeLa shN and HeLa shR cells following phenylhydrazine (PHZ) stimulation and overexpression of HBD. We observed that overexpression of HBD in HeLa shN cells showed significant downregulation of HBD by PHZ stimulation in a time-dependent manner. Moreover, overexpressed HBD HeLa shR cells were unable to induce downregulation of HBD with PHZ treatment, as estimated by Western blot analysis (Figure 7). Taken together, our data demonstrate that REG*γ* induces the degradation of HBD under oxidative stress.

## 4. Discussion

Hemoglobin is a major protein of erythrocytes (RBCs) responsible for the transport of oxygen (O_2_). To maintain maximum O_2_-carrying capacity, Hb must be kept under reduced condition known as ferrous (Fe^2+^) bound state. The oxidation of Hb from ferrous to ferric states is accelerated by reactive oxygen species (ROS) such as superoxide [19] and hydroxyl free radical [20]. The Hb in the aging erythrocytes (RBCs) is mainly scavenged by the CD163 scavenger receptor pathway through monocytes or macrophages [21, 22], while abnormal Hb in the intracellular system is degraded through the proteasomes [23]. In fact, physiological and pathological stresses on the RBCs accelerate the eryptosis period which possibly led to Hb damage. Further fine-tuning hemoglobin function are oxygen transport, carbon dioxide transport, iron-rich substances, reactive oxygen species (ROS) accumulation, and oxidative damage [19, 20]. Hb binding to the erythrocyte cell membrane has been implicated in senescence and the consequent targeting of the red cell for removal from the circulation by macrophages via phagocytosis. Recent studies demonstrate that oxidized Hb degradation is contributed in the RBC by proteasome and its activators. However, the precise roles of proteasomes in the Hb and Hb subunit degradations within RBCs are still a matter of debate. In the present study, for the first time, the results reported herein clearly demonstrate that REG*γ* mediates degradation of Hb and HBD in hematopoietic tissues and nonerythroid cells. Specifically, protein level of Hb was detected in hematopoietic tissues from neonatal mice, supporting a broader role of REG*γ* in the regulation of hemoglobin.

CD163 is an Hb scavenger receptor exclusively expressed in the cell's monocyte/macrophage system. Resident tissue macrophages contain the highest levels of CD163, most notably Kupffer cells in the liver and macrophages within the bone marrow and spleen red pulp [24]. Moreover, the aging erythrocytes are susceptible to recognition by phagocytes and subsequent phagocytosis in the spleen, liver, and bone marrow. Therefore, liver, bone marrow, and spleen tissues are the major sites expressing CD163 and removing aging erythrocytes. Our results showed that CD163 mRNA level was notably decreased in liver, spleen, and bone marrow hemopoietic tissues from REG*γ* knockout mice. Thus, REG*γ* positively regulates CD163 gene expression in the hemopoietic tissues, which suggests that REG*γ* selectively mediated Hb degradation through Hb scavenger receptor pathway. Yet the regulatory mechanisms deserve further studies.

The study of Hb subunit degradation provides new insights to research in Hb degradation. The finding that HBD is a target of REG*γ* demonstrates a new layer of HB regulation. Despite that HBD variant (HbA_2_ or α_2_*δ*_2_) is a minor component (2-3%) in the circulating red blood cells, it has important physiological and pathological roles given its unusual elevation in *β*-thalassemia as a useful clinical diagnostic [24]. HBD also shows high level of gene sequence conservation, possibly due to a regulatory role in the fetal-to-adult switch [25]. We validated degradation of HBD by the REG*γ* proteasome with dynamic protein stability assays. These results promoted us to determine the protein level of HBD as well as cellular location with or without REG*γ* overexpression in HeLa cells. We conclude that REG*γ* appears to be an important factor in the degradation of HBD.

Several studies have shown an apparently increasing rate of proteolysis in RBCs during oxidative stress. Hb is the major RBC protein, which plays a critical role in the modification and proteolytic degradation during oxidative stress. Selectively, oxidative-modified Hb degradation in an ATP- and ubiquitin-independent manner is almost 60–70% due to oxidative damage to the 26S system [26]. Recently, our laboratory has discovered that the intensity of REG*γ* binding to proteasome was enhanced by mild or severe oxidative stress [7], suggesting that phenylhydrazine-mediated degradation of HBD may be due to strengthened interactions between REG*γ* and 20S proteasome. The strong oxidative feature of phenylhydrazine has been previously shown to probe hemoglobin oxidative damage. In support of this hypothesis, significant HBD degradation has been observed in phenylhydrazine-treated HeLa shN cells in a time-dependent manner. Therefore, our results indicate that REG*γ* promotes HBD degradation under oxidative stress.

In conclusion, our study substantiates that REG*γ* selectively mediates the degradation of Hb and HBD. In fact, the important role of REG*γ*-mediating Hb and HBD degradation demonstrates an additional pathway for the breakdown of Hb and HBD and provides certain theoretic basis for preventing and curing Hb-related diseases. These results suggest REG*γ* could be a promising drug target. However, further work is necessary to understand the biological significance and role of REG*γ* in selective degradation of Hb and HBD.
